# Distribution of avian influenza viruses according to environmental surveillance during 2014–2018, China

**DOI:** 10.1186/s40249-021-00850-3

**Published:** 2021-05-06

**Authors:** Hong Bo, Ye Zhang, Li-Bo Dong, Jie Dong, Xi-Yan Li, Xiang Zhao, Zi Li, Yue-Long Shu, Da-Yan Wang

**Affiliations:** 1 National Institute for Viral Disease Control and Prevention, Chinese Center for Disease Control and Prevention; WHO Collaborating Center for Reference and Research On Influenza; Key Laboratory for Biosafety, National Health Commission, Beijing, China; 2 Public Health School (Shenzhen), Sun Yat-Sen University, Guangzhou, China

**Keywords:** Avian influenza virus, Live poultry market, Environment, Surveillance

## Abstract

**Background:**

Recurrent infections of animal hosts with avian influenza viruses (AIVs) have posted a persistent threat. It is very important to understand the avian influenza virus distribution and characteristics in environment associated with poultry and wild bird. The aim of this study was to analyze the geographic and seasonal distributions of AIVs in the 31 provinces, municipalities and autonomous region (PMA) of China, compare the AIVs prevalence in different collecting sites and sampling types, analyze the diversity of AIVs subtypes in environment.

**Methods:**

A total of 742 005 environmental samples were collected from environmental samples related to poultry and wild birds in different locations in the mainland of China during 2014–2018. Viral RNA was extracted from the environmental samples. Real-time RT-PCR assays for influenza A, H5, H7 and H9 subtypes were performed on all the samples to identify subtypes of influenza virus. The nucleic acid of influenza A-positive samples were inoculated into embryonated chicken eggs for virus isolation. Whole-genome sequencing was then performed on Illumina platform. SPSS software was used to paired t test for the statistical analysis. ArcGIS was used for drawing map. Graphpad Prism was used to make graph.

**Results:**

The nucleic acid positivity rate of influenza A, H5, H7 and H9 subtypes displayed the different characteristics of geographic distribution. The nucleic acid positivity rates of influenza A were particularly high (25.96%–45.51%) in eleven provinces covered the Central, Eastern, Southern, Southwest and Northwest of China. The nucleic acid positivity rates of H5 were significantly high (11.42%–13.79%) in two provinces and one municipality covered the Southwest and Central of China. The nucleic acid positivity rates of H7 were up to 4% in five provinces covered the Eastern and Central of China. The nucleic acid positivity rates of H9 were higher (13.07%–2.07%) in eleven PMA covered the Southern, Eastern, Central, Southwest and Northwest of China. The nucleic acid positivity rate of influenza A, H5, H7 and H9 showed the same seasonality. The highest nucleic acid positivity rates of influenza A, H5, H7, H9 subtypes were detected in December and January and lowest from May to September. Significant higher nucleic acid positivity rate of influenza A, H5, H7 and H9 were detected in samples collected from live poultry markets (LPM) (30.42%, 5.59%, 4.26%, 17.78%) and poultry slaughterhouses (22.96%, 4.2%, 2.08%, 12.63%). Environmental samples that were collected from sewage and chopping boards had significantly higher nucleic acid positivity rates for influenza A (36.58% and 33.1%), H5 (10.22% and 7.29%), H7(4.24% and 5.69%)and H9(21.62% and 18.75%). Multiple subtypes of AIVs including nine hemagglutinin (HA) and seven neuraminidase (NA) subtypes were isolated form the environmental samples. The H5, H7, and H9 subtypes accounted for the majority of AIVs in environment.

**Conclusions:**

In this study, we found the avian influenza viruses characteristics of geographic distribution, seasonality, location, samples types, proved that multiple subtypes of AIVs continuously coexisted in the environment associated with poultry and wild bird, highlighted the need for environmental surveillance in China.

**Graphic Abstract:**

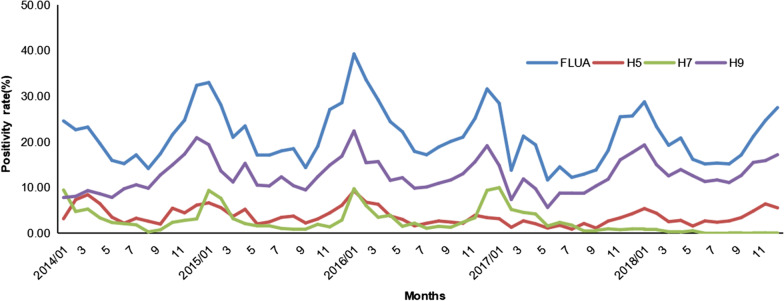

**Supplementary Information:**

The online version contains supplementary material available at 10.1186/s40249-021-00850-3.

## Background

AIVs were first reported in 1878 [1] in Italy and were subsequently isolated from chickens in 1934 [2]. AIVs are categorized into two pathotypes according to their virulence in chickens: low-pathogenic and highly pathogenic AIVs [3, 4]. Of the 16 HA subtypes that have been identified in birds, 5 HA subtypes of AIVs (H5N1, H5N6, H5N8, H6N1, H7N9, H7N2, H7N3, H7N7, H9N2, H10N7, and H10N8) are known to cause human infections [5, 6], among which the H5 subtype has a nearly global distribution in birds. Moreover, the H7N9 subtype widely circulates and had rapidly evolved in LPMs in China especially from 2013 to 2018 [7, 8]. Furthermore, continuous reassortments among AIV strains have increased the risk of a pandemic.

Many studies have investigated the demographic and ecological risk factors associated with the effective transmission of AIVs. LPMs play an important role in human infection with AIVs. Bird transport between LPMs affected the emergence of H7N9 in Eastern China, and the closure of LPMs reduced the incidence of human infection with AIVs [9]. Furthermore, one survey found that 80% of households that purchased poultry from LPMs had an increased risk of poultry-to-human infection [10]. LPMs are particularly common in Southern China, and many subtypes, such as H9, H5, and H6, have been enzootic in poultry in China since the mid-1990s [11]. However, environmental factors of LPM, such as temperature, humidity, and feeding conditions, play important roles in AIV survival and infectivity [12].

Environmental surveillance related poultry and wild bird was established in China in 2009. Samples are collected each month by each province. In the present study, we analyzed the environmental samples collected from 2014 to 2018 as part of this programme to examine the geographic and seasonal distributions of AIVs in China and how these vary with regard to sites and sample types to determine environmental risk factors.

## Methods

### Environmental sample collection sites

Samples were collected from 2014 to 2018 by Chinese National Influenza Surveillance Network laboratories. Based on the national surveillance guidelines, at least 40 samples per month were collected from each of the 31 PAM in China located in seven regions (Eastern, Southern, Central, Northern, Northwest, Southwest, and Northeast of China), providing a total of 742,005 samples. These samples were obtained from a range of sites, including LPMs, poultry farms, backyards, slaughterhouses and wild bird habitats. These samples were obtained from a variety of poultry-related materials, including poultry faeces, drinking water, sewage, and swabs from poultry cages, feathers, etc. Each sample was maintained in viral transport medium and immediately transported at a low temperature to the nearest Chinese National Influenza Surveillance Network laboratory.

### Environmental sample collection

Faeces or swab samples were put into 5 ml Hank’s medium containing 0.5% bovine albumin, ampicillin (2 × 10^6^ IU/L), streptomycin (200 mg/L), polymyxin B (2 × 10^6^ IU/L), gentamicin (250 mg/L), mycin (0.5 × 10^6^ IU/L), oxygen hydrochloride floxacin (60 mg/L), and sulfamethoxazole (200 mg/L). For liquid samples (drinking water, sewage), 5-ml liquid was collected. The samples were sent to a local network laboratory within 48 h and stored at 4 ℃. The samples were mixed thoroughly, centrifuged at 3000 rpm for 10 min, and the supernatants were aliquoted into three tubes. Each tube contained about 1.5 ml of the sample; one was designated for nucleic acid identification by a local network laboratory, one was designated for virus isolation, and one was transported to the Chinese National Influenza Center and then stored at −80 ℃ for further test.

### RNA extraction

Viral RNA was extracted from each of the collected samples using a QIAsymphony RNA Kit (931636; Qiagen, Hilden, Germany) with a QIAsymphony SP instrument (Qiagen) according to the manufacturer's instructions.

### Real-time RT-PCR

Real-time RT-PCR assays for influenza A, H5, H7, and H9 subtypes were performed on all the samples, with primer and probe sets provided in the Chinese National Influenza Surveillance Guidelines (Additional file 1: Table S1). The reactions were carried out using an AgPath-ID™ One-Step RT-PCR Kit (4387422; Ambion®) under the following cycling conditions: 10 min at 45 ℃; 10 min at 95 ℃; 40 cycles of 15 s at 95 ℃; and 45 s at 60 ℃. The positive control contained all the reaction components and RNA of influenza A, and H5, H7, and H9 subtypes. The negative control contained all the reaction components and H_2_O as the template.

### Virus isolation

Virus isolation was performed on the influenza A-positive samples detected by Real-time RT-PCR. The samples were inoculated into the allantoic cavity of 9 to 10-day-old embryonated chicken eggs; the eggs were incubated at 37 ℃ for 48 h and chilled at 4 ℃ overnight. The allantoic fluid was then harvested, and a haemagglutination assay was performed using 1% turkey red blood cells to determine the titer of harvested viruses [13].

### Next-generation sequencing

Virus total RNA was extracted by a MagMAX™ Viral/Pathogen Nucleic Acid Isolation Kit (cat# 42352, Applied Biosystems). The RNA was subjected to reverse transcription and amplification using the SuperScript™ III One-Step RT-PCR System with Platinum™ Taq High Fidelity DNA Polymerase (cat#: 12574035, Invitrogen). The DNA was purified by a MagMax Core Nucleic Acid Purification Kit (cat# 1903031, Thermo Fisher Scientific). The DNA library was prepared using Nextera XT DNA Preparation Kits (cat#FC-131-1096, Illumina). Whole-genome sequencing was then performed on MiSeq high-throughput sequencing platform and Nextseq platform (Illumina, Inc., San Diego, CA, USA), and the data were analysed using CLC Genomics Workbench software.

### Statistical analysis

The statistical software SPSS version 25 (IBM Corp, New York, USA) was used to data statistical analysis. A paired *t* test was performed to analyze the significant difference. *P* < 0.05 was considered significant.

### Map plotting

ArcGIS version 10.7 (Environmental Systems Research Institute, RedLands, USA) was used to draw the map. Different colors represented the range of positivity rate on the map.

### Graph making

Graphpad Prism5 (Graphpad software Inc, San Diego, USA) was used to process the data and made graph.

## Results

### Geographic distributions of avian influenza viruses

**Fig. 1:**
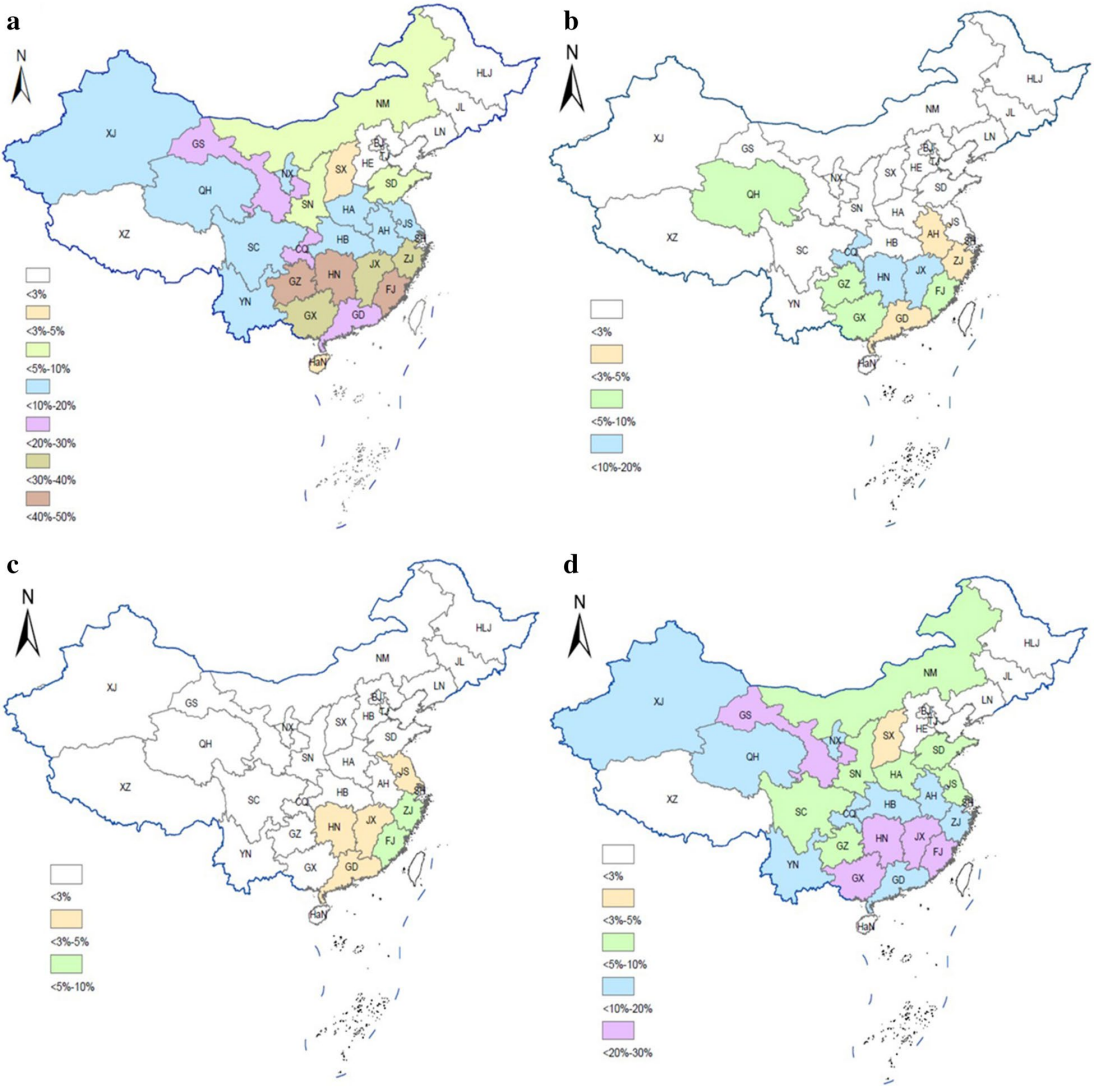
Geographical distributions of the nucleic acid positivity rates of influenza A, H5, H7 and H9 subtypes. **a**–**d** indicates the nucleic acid positivity rates of influenza A, H5, H7 and H9 subtypes on average in 31 PMA during 2014–2018, respectively. Different colours represent the corresponding range of positivity rates. Abbreviations: AH, Anhui; BJ, Beijing; CQ, Chongqing; FJ, Fujian; GD, Guangdong; GS, Gansu; GX, Guangxi; GZ, Guizhou; HA, Henan; HB, Hubei; HE, Hebei; HLJ, Heilongjiang; HN, Hunan; HaN, Hainan; JL, Jilin; JS, Jiangsu; JX, Jiangxi; LN, Liaoning; NM, Inner Mongolia; NX, Ningxia; QH, Qinghai; SC, Sichuan; SD, Shandong; SH, Shanghai; SX, Shanxi; SN, Shaanxi; TJ, Tianjin; XJ, Xinjiang; XZ, Tibet; YN, Yunnan; ZJ, Zhejiang. PAM: Provinces, municipalities and autonomous regions

Based on RT-PCR, the positivity rate for influenza A was 22.57% (Fig. 1a). The positivity rates were particularly high (25.96%–45.51%) in 11 provinces in five of the seven regions tested: Central China (Hunan provinces), Eastern China (Fujian, Jiangxi and Zhejiang provinces), Southern China (Guangxi Autonomous Region), Southwest China (Chongqing Municipality and Guizhou Province), and Northwest China (Gansu Province).

Among the influenza A viruses detected, the H5 subtype was detected in 3.89% of the samples (Fig. 1b). The positivity rates were significantly high (11.42%–13.79%) in two provinces and one municipality in two regions: Southwest China (Chongqing Municipality), Central China (Hunan and Jiangxi provinces).

The positivity rate for the H7 subtype was 3.41% (Fig. 1c), with rates of up to 4% being detected in two regions: Eastern China (Jiangsu, Fujian, Zhejiang and Jiangxi provinces) and Central China (Hunan Province).

The positivity rate for the H9 subtype is 13.07% (Fig. 1d), with 11 PAM displayed high positivity rates (13.07%–27.07%). These regions included Southern China (Guangdong Province and Guangxi Autonomous Region), Eastern China (Zhejiang, Anhui, Jiangxi and Fujian provinces), Central China (Hunan Province), Southwest China (Yunnan Province and Chongqing Municipality) and Northwest China (Gansu Province and Xinjiang Autonomous Region). The nucleic acid positivity rates of influenza A H5, H7, and H9 subtypes in different regions were shown in Fig. 1 and Additional file 1: Table S2.

### Seasonality of avian influenza viruses in environments

**Fig. 2:**
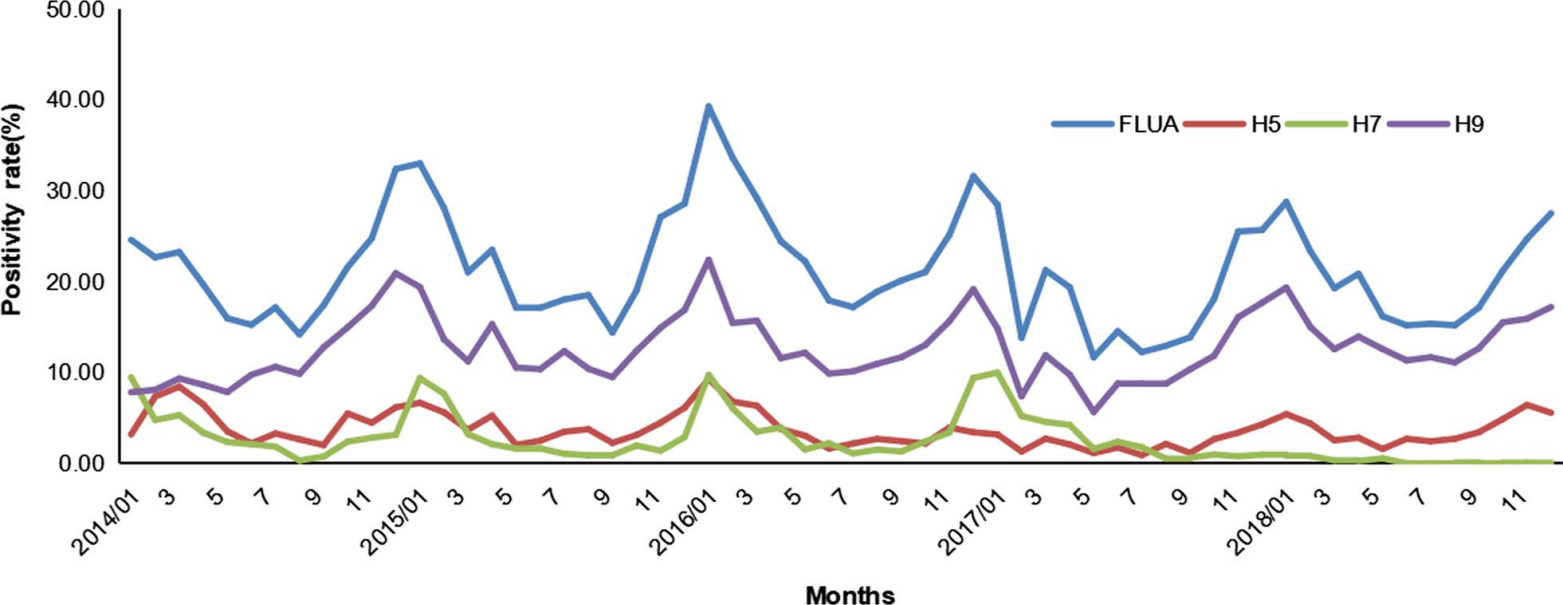
Monthly nucleic acid positivity rates of influenza A, H5, H7 and H9 subtypes. The blue line represents the nucleic acid positivity rate of influenza A; the red line represents the nucleic acid positivity rate of H5 subtype; the light green line represents the nucleic acid positivity rate of H7 subtype; the purple line represents the nucleic acid positivity rate of H9 subtype

The monthly nucleic acid positivity rates of influenza A, H5, H7 and H9 subtypes in the poultry-related environmental samples were shown in Fig. 2 and Additional file 1: Table S3. The positivity rates of influenza A, H5, H7 and H9 subtypes showed obvious seasonality and were highest in December and January and lowest from May to September.

### Variations of positivity rates of influenza A, H5, H7 and H9 subtypes among the sampling sites

During 2014–2018, samples with the highest positivity rates of influenza A, H5, H7 and H9 were collected from LPMs (30.42%, 5.59%, 4.26%, 17.78%), followed by slaughterhouses (22.96%, 4.2%, 2.08%, 12.63%), respectively. In contrast, poultry farms, backyards and wild bird habitats had influenza A positivity rates of 3.26%, 3.44%, and 1.1% respectively, while the H5 and H7 positivity rates were all less than 1%. Poultry farms, backyards, and wild bird habitats had H9 positivity rates of 1.98%, 1.42%, and 0.25%. The statistical analysis indicated that LPMs and slaughterhouses were associated with significantly higher positivity rates of AIVs than all other sites during the study period (*P* < 0.05; Table 1).

**Table 1:** Influenza A, H5, H7 and H9 nucleic acid positivity rates of different sites

Type/subtype	Positivity rate (%)				
	(Number of positive samples/Number of samples collected)				
Year	LPM	Slaughterhouse	Backyard	Poultry farm	Wild bird habitat
Influenza A					
2014	30.61 (12050/39365)	16.67 (372/2232)	1.69 (82/4847)	2.31 (242/10458)	4.12 (86/2086)
2015	30.2 (14245/47174)	19.16 (396/2067)	4.23 (185/4373)	2.85 (270/9470)	0.53 (11/2080)
2016	35.24 (17910/50882)	25.04 (475/1897)	5.4 (285/5274)	4.69(546/11649)	0.35(7/2003)
2017	25.98 (20620/79377)	23.70 (720/3039)	2.83 (341/12036)	3.33 (508/15251)	0.33 (10/3076)
2018	30.06 (14428/47999)	30.22 (727/2406)	3.04 (246/8098)	3.10 (332/10705)	0.19 (5/2683)
Mean ± SD	30.42 ± 3.28	22.96 ± 5.28	3.44 ± 1.41	3.26 ± 0.88	1.1 ± 1.69
A/H5					
2014	7.2 (2833/39365)	3.18 (71/2232)	0.21 (10/4847)	0.73 (76/10458)	0 (0/2086)
2015	5.94 (2802/47174)	3.87 (80/2067)	0.8 (225/4373)	0.29 (27/9470)	0.1 (2/2080)
2016	6.01(3057/50882)	5.01 (95/1897)	0.36 (19/5274)	0.29(34/11649)	0.1(2/2003)
2017	3.24 (2572/79377)	2.47 (75/3039)	0.19 (23/12036)	0.19 (29/15251)	0 (0/3016)
2018	5.54 (2658/47999)	6.48 (156/2406)	0.3 (24/8098)	0.45 (48/10705)	0 (0/2683)
Mean ± SD	5.59 ± 1.45	4.2 ± 1.58	0.37 ± 0.24	0.39 ± 0.21	0.04 ± 0.05
A/H7					
2014	5.43 (2139/39365)	0.76 (17/2232)	0.52 (25/4847)	0.71 (74/10458)	1.39 (29/2086)
2015	4.73 (2231/47174)	2.18 (45/2067)	0.8 (35/4373)	0.04 (4/9470)	0 (0/2080)
2016	5.57 (2832/50882)	3.22 (61/1897)	1.31 (69/5274)	0.25 (29/11649)	0.05(1/2003)
2017	5.15 (4090/79377)	2.57 (78/3039)	0.75 (90/12036)	1.36 (207/15251)	0 (0/3016)
2018	0.42 (200/47999)	1.66 (40/2406)	0.14 (11/8098)	0.05 (5/10705)	0 (0/2683)
Mean ± SD	4.26 ± 2.17	2.08 ± 093	0.7 ± 0.42	0.48 ± 0.56	0.28 ± 0.6
A/H9					
2014	15.68 (6173/39365)	8.47 (189/2232)	0.8 (39/4847)	1.1 (115/10458)	0.48 (10/2086)
2015	18.04 (8509/47,174)	9.82 (203/2067)	2.74 (120/4373)	1.19 (113/9470)	0.24 (5/2080)
2016	19.51 (9915/5088)	13.86 (263/1897)	3.3 (174/5274)	1.48 (172/11649)	0.2 (4/2003)
2017	15.05 (11950/79377)	14.61 (444/3039)	1.37 (165/12036)	1.24 (189/15251)	0.2 (6/3016)
2018	20.61 (9893/47999)	16.38 (394/2406)	1.68 (136/8098)	2.10 (225/10705)	0.112 (3/2683)
Mean ± SD	17.78 ± 2.39	12.63 ± 3.34	1.98 ± 1.02	1..42 ± 0.4	0.25 ± 0.14

Real-time RT-PCR method is used to screen for influenza A, H5, H7 and H9 subtypes in the samples. The positivity rate is defined as number of positive samples/number of sample collected

LPM, Live poultry market, SD, Standard deviation

### Variations of the positivity rates of influenza A, H5, H7 and H9 subtypes among different sample types

Environmental samples that were collected from sewage and chopping boards had significantly higher positivity rates for influenza A (36.58% and 33.1%), H5 (10.22% and 7.29%), H7 (4.24% and 5.69%) and H9 (21.62% and 18.75%) than those collected from faeces, cages, and feeding troughs (*P* < 0.05). Furthermore, the positivity rates of H9 in samples originating from sewage and chopping boards were significantly higher than those of H5 and H7 subtypes (*P* < 0.05; Table 2).

**Table 2:** Influenza A, H5, H7 and H9 nucleic acid positivity rates of different sample types

Type/subtype	Positivity rate (%)				
	(Number of positive samples/Number of samples collected)				
Year	Sewage	Chopping board	Feeding trough	Cage	Faeces
Influenza A					
2014	37.31 (2819/7556)	33.49 (2373/7085)	26.15 (2138/8175)	17.81 (2538/14252)	12.92 (2617/20259)
2015	39.03 (3025/7750)	33.74 (3217/9535)	28.19 (2300/8158)	19.76 (3196/16174)	15.05 (3333/22141)
2016	40.17 (3774/9394)	40.25 (4558/11324)	27.64 (2797/10121)	24.01 (4013/16711)	18.79 (4503/23962)
2017	31.03 (4559/14692)	27.03 (4712/17433)	22.48 (3450/15347)	18.19 (4715/25919)	12.34 (4398/35648)
2018	35.37 (3190/9020)	30.99 (3074/9919)	23.73 (2613/11011)	19.47 (3122/16035)	13.87 (3237/23347)
Mean ± SD	36.58 ± 3.59	33.1 ± 4.82	25.64 ± 2.46	19.85 ± 2.46	14.59 ± 2.56
A/H5					
2014	14.56 (1100/7556)	7.86 (2373/7085)	7.38 (2138/8175)	2.15 (2538/14252)	1.9 (385/20259)
2015	12.12 (939/7750)	7.07 (674/9535)	6.06 (494/8158)	2.35 (380/16,174)	1.89 (418/22,141)
2016	9.59 (901/9394)	9.04 (1024/11324)	4.48 (453/10121)	2.59 (433/16711)	2.18 (522/23962)
2017	5.64 (828/14692)	4.37 (762/17433)	2.42 (372/15347)	1.48 (383/25919)	0.99 (353/35648)
2018	9.20 (830/9020)	8.11 (804/9919)	3.54 (390/11011)	2.56 (411/25919)	1.77 (413/23347)
Mean ± SD	10.22 ± 3.34	7.29 ± 1.77	4.78 ± 1.97	2.23 ± 0.45	1.75 ± 0.44
A/H7					
2014	4.74 (358/7556)	6.68 (473/7085)	3.61 (295/8175)	3.96 (564/14252)	2.35 (477/20259)
2015	4.25 (329/7750)	6.12 (584/9535)	3.33 (272/8158)	3.52 (569/16174)	2.81 (622/22141)
2016	5.8 (545/9394)	8.9 (1008/11324)	2.93 (297/10121)	4.3 (718/16711)	3.19 (765/23962)
2017	5.73 (842/14692)	6.31 (1100/17433)	4.06 (624/15347)	3.65 (946/25919)	2.59 (924/35648)
2018	0.68 (61/9020)	0.44 (44/9919)	0.27 (30/11011)	0.28 (45/16035)	0.28 (66/23347)
Mean ± SD	4.24 ± 2.09	5.69 ± 3.13	2.84 ± 1.49	3.14 ± 1.62	2.24 ± 1.14
A/H9					
2014	19.1 (1443/7556)	17.12 (1213/7085)	15.40 (1259/8175)	9.12 (1300/14252)	5.98 (1212/20259)
2015	24 (1860/7750)	19.17 (1828/9535)	19.48 (1589/8158)	12.06 (1951/16174)	8.05 (1782/22141)
2016	23.32 (2191/9394)	21.3 (2412/11324)	16.47 (1667/10121)	13.44 (2246/16711)	9.32 (2234/23962)
2017	18.48 (2715/14692)	14.34 (2505/1433)	13.12 (2013/15347)	11.4 (2954/25919)	6.36 (2266/35648)
2018	23.16 (2089/9020)	21.82 (2164/9919)	14.52 (1599/11011)	14.72 (2360/16035)	8.89 (2075/23347)
Mean ± SD	21.62 ± 2.6	18.75 ± 3.09	15.80 ± 2.39	12.14 ± 2.12	7.72 ± 1.49

Real-time RT-PCR method is used to detect influenza A, H5, H7 and H9 subtypes in different sample types. The positivity rate is defined as number of positive samples/number of sample collected

SD, Standard deviation

### Multiple subtypes of influenza A viruses were detected in poultry-related environments

**Fig. 3:**
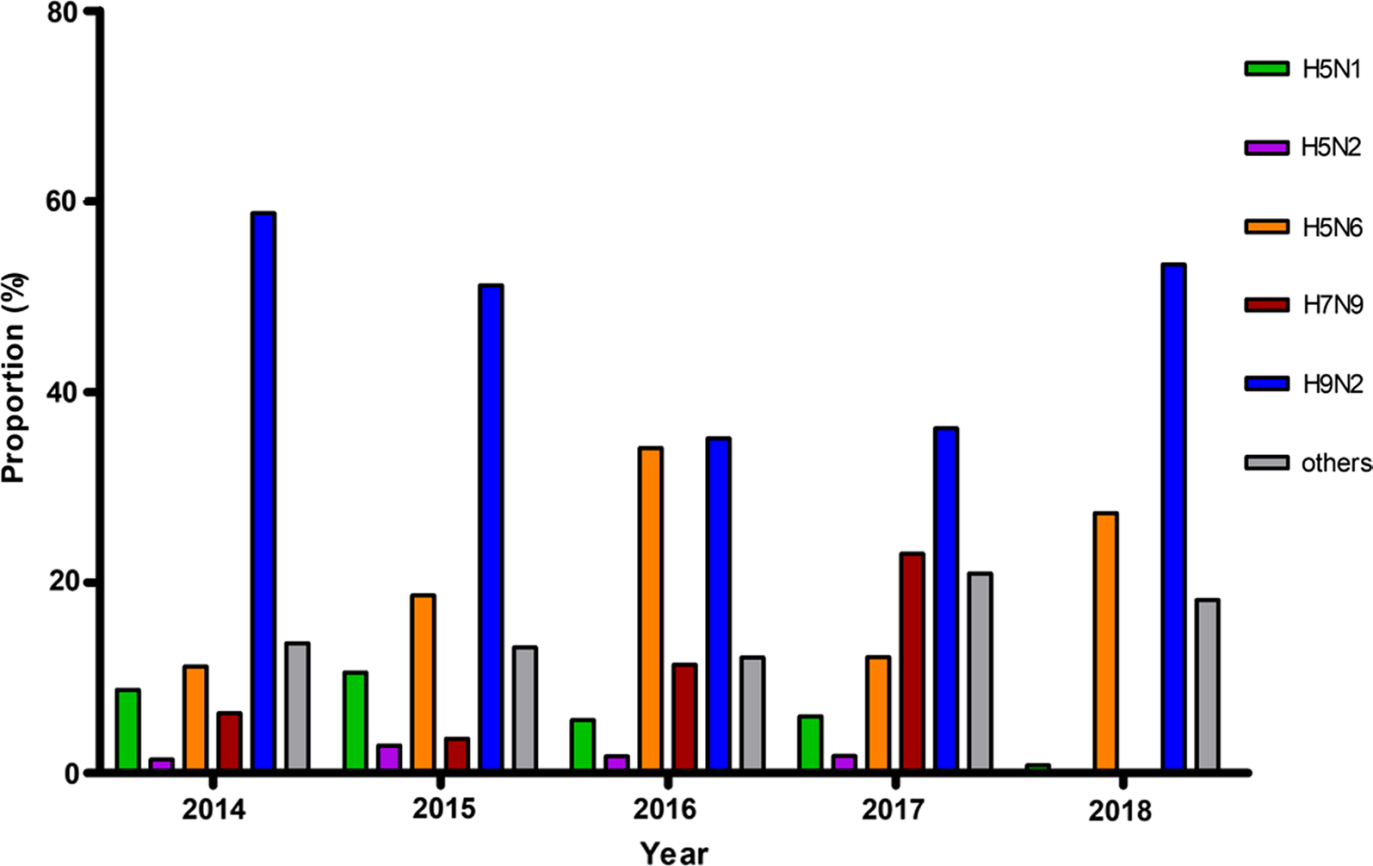
Proportions of H5N1, H5N6, H7N9, H9N2 AIVs and other virus subtypes during 2014–2018. The X axis represents different years, and the Y axis represents proportion of different subtypes AIVs (%). The proportion is defined as the number of different AIVs subtypes/number of total viruses (%). Abbreviations AIVs: avian influenza viruses

In total, nine HA subtypes and seven NA subtypes of avian influenza viruses were isolated including the HA subtypes H1, H3, H4, H5, H6, H7, H9, H10, and H11, and the NA subtypes N1, N2, N3, N6, N7, N8, and N9. In general, the H5, H7, and H9 subtypes of influenza A virus accounted for the majority of avian influenza viruses isolated from the environmental samples (Additional file 1: Table S4). The proportion of H9N2 avian influenza viruses took a proportion of 46.90% among all the subtypes. The proportion of H5N6 and H5N1 AIVs were 20.66% and 6.31%. The proportion of H7N9 AIVs was 8.9%. The proportion of other subtypes were less than 0.5% except for H3N2 (6.15%), H6N6 (4.43%), and mixture subtypes (1.58%). The H9N2, H5N6 and H7N9 AIVs displayed obviously different proportions from 2014 to 2018. The proportion of subtype H5N6 AIVs increased more than threefold from 11% in 2014 to 34% in 2016, then decreased to 12.14% in 2017. The H5N1 AIVs showed a declining trend from 2014 to 2018. The proportion of H7N9 AIVs reached 23% in 2017, which was approximately four times that in 2014 (6%) and decreased to 0.26% in 2018. The proportion of H9N2 AIVs showed a decreasing from 2014 to 2017, with proportions of 59%, 51%, 35% and 36%, respectively then increased to 53.36% in 2018 (Fig. 3 and Additional file 1: Table S4).

## Discussion

Based on five-year environmental surveillance data, we analysed the geographical distributions of influenza A, H5, H9 and H7 subtypes in AIVs related environment. The geographical distribution of H7 subtype mainly occurred in the Yangtze and Pearl River deltas and a few adjacent provinces. It was reported that these regions were the sources of newly emerged H7N9 human infections during 2013–2017 [11]. Poultry is the major source of H7N9 infections in humans [14]. Our study indicates that the H7 subtype persists in the environment in these regions and may be associated with human infection and virus evolution. The geographical distribution of H5 was mainly located in Southern China. H5 has been widely circulating among poultry in China since 2004. The highly pathogenic avian influenza H5 subtype has evolved multiple clades and subclades [15]. Though control measures were carried out, LPMs and other places are still providing the opportunity for transmission and evolution of H5 subtype, birds and poultry still carry and transmit the H5 AIVs. Compared with the H5 and H7 subtypes, the H9 subtype geographic distribution was nationwide, and it was especially prevalent in the southern and western regions of China. The first H9N2 subtype was isolated in 1992, and since then, H9N2 has become the predominant subtype in poultry [16]. Live poultry trading and feeding patterns have caused H9N2 to become prevalent in different regions of China [17]. The wide distribution of H9 subtype provides the opportunity for avian virus reassortment.

Our results indicated that the prevalence of AIVs varied seasonally, with higher positivity rates of H5, H7 and H9 subtypes in late winter (December) and early spring (January) than in summer and autumn. Several studies have found that minor fluctuations in temperature, pH or salinity in aquatic habitats may enhance or diminish the persistence and infectivity of AIVs [18]. AIV transmission is promoted under cool and dry conditions [19]. Our results were consistent with those of current studies and showed that AIVs can survive well in winter and early spring with higher rate. In our study, LPMs and sewage were proved to be environmental risk factors of AIVs. The samples collected from LPMs displayed the highest positivity rates for the H5, H7 and H9 subtypes, which is consistent with previous studies. In China, a large number of poultry is traded through LPMs [20] which are known to be major sources of AIVs causing significant public health concerns. Poultry market closure is still an effective measure to prevent avian influenza infects humans or poultry [21]. We recommended that LPMs should be managed strictly. Some studies have reported that influenza viruses are waterborne pathogens that have the capacity to infect a wide variety of hosts and undergo genetic reassortment [22] In our study, we investigated five kinds of samples related to poultry feeding, sale, and slaughter. The results indicated that sewage may carry a large number of AIVs and transmit the virus among poultry.

Our studies demonstrated virus subtypes diversity in environment-related poultry and wild bird. In our study, virus isolation results indicated that the H5 subtype AIVs continuously existed in environmental samples in China and the proportion of H5N1 subtype AIVs viruses decreased while the proportion of H5N6 subtype AIVs viruses increased dramatically during 2014–2017. In 2018, the proportion of H5N1 subtype AIVs significantly declined to 0.8%. Liu et al. have reported the epidemiology and evolution characteristics of H5 AIVs subtype in poultry since 2007. The H5 subtype was almost exclusively H5N1 subtypes before 2012. Since 2014, H5N6, H5N2 and H5N8 subtypes AIVs emerged and H5N6 subtype became predominantly in China. In 2018, 78.0% and 21.8% of all AIV isolates were identified as H5N6 and H5N2 subtypes respectively [23]. In accordance with these studies, our data also revealed that the N subtype of H5 shifted from N1 to N6 during 2014–2018 in environment related AIVs. Some control strategies were carried out in order to prevent the H5 spread, including mass vaccination in poultry in China. Vaccine strain of H5 subtype was updated every few years to combat the vaccine escape mutants. Nine vaccine strains (N-28, Re-1,4,5,6.7.8,11,12) were used during 2005–2018 [23, 24]. The clade 7.2 of H5 AIVs were almost eliminated in chickens since 2014 largely due to mass vaccination while the clade 2.3.2 and 2.3.4 of H5 AIVs were prevalent in waterfowl and also identified in wild birds. The reason was due to the following points. Firstly, vaccination can lead to silent AIV infections and accelerate virus transmission and diversification. Second, low vaccination coverage and immune response to the vaccine reduced the vaccination effect in waterfowl [24]. Third, wild bird and waterfowl can transmit the virus each other and vaccination cannot block the transmission. Fourth, vaccine strains need to be updated accurately. These reasons may explain why H5N6 subtype AIVs substituted H5N1 subtype AIVs and became the dominant in the environment.

The avian influenza A(H7N9) virus infection was first detected in humans in March 2013. Until March 2018, a total of 1567 laboratory-confirmed human cases have been reported to WHO [25]. Some studies reported that poultry-related environments were contaminated, and poultry were infected with H7N9 AIV s in the regions where H7N9 AIV s emerged [26]. Jiang et al. reported the H7N9 AIV s prevalence in poultry and evaluated the H7N9 AIV s vaccine protection efficacy in China during 2013–3018. H7N9 AIVs positivity rate varied from 2013 to 2018 in poultry, 0.25% in 2013,0.76% in 2014,0.88% in 2015, 0.41% in 2016, 0.97% in 2017 and 0.00% in 2018. Large scale administration of H7-Re1vaccine made the H7N9 prevalent decrease sharply in poultry [27]. In our study, we also found that the percentage of H7N9 subtype varied in the environment, especially in 2017 (the percentage was up to 23%) while the percentage of H7N9 decrease to 0.26% in 2018. The results indicated that the spread of the H7N9 AIV s in poultry and the environment has been effectively controlled and prevented human infection.

Currently, H9N2 remained highly prevalent in poultry. BJ/94-like and G1-like lineage of H9N2 AIVs have been mainly prevalent in China since the mid-1990s [28]. Since 1998, vaccination was carried out in poultry and vaccine strains were updated. However, H9N2 AIVs persisted in chickens due to antigenic drift and low vaccination coverage rate [29]. Our results indicated that H9N2 AIVs remained the highest percentage in environment samples. H9N2 AIVs prevalence in poultry and environment provided the chance for zoonotic transmission and virus reassortant.

Our study demonstrated the avian influenza virus characteristics in the environment related to poultry and wild birds in China. There were limitations in our study. Firstly, considering that there are many LPMs in China, especially in rural areas, the data in our study was collected from the environment of a small number of LPMs in China, there may be bias in the representativeness of the data. Secondly, the data only reflected the general features instead of individual characteristics, although the environment was related to waterfowl (duck and goose), domestic fowl (chicken) and wild bird in our study.

## Conclusions

Our findings indicate that environments associated with poultry may contribute to the transmission of AIVs. The widespread and persistent circulation of avian influenza viruses in China increases the risk of zoonotic transmission and encourages the timely monitoring of changes in AIVs. Long-term control strategies and early interventions need to be developed for AIV outbreaks.

## Supplementary Information

**Additional file 1: Table S1.** Primer and probe sequences used in the detection of influenza virus. **Table S2.** The average nucleic acid positivity rate of influenza A, H5, H7 and H9 in different provinces, municipalities and autonomous regions. Table S2-a, S2-b, S2-c, S2-d indicated the average nucleic acid positivity rate of influenza A, H5, H7 and H9 respectively. **Table S3.** The monthly nucleic acid positivity rate of influenza A, H5, H7 and H9 in environmental samples related poultry during 2014–2018. **Table S4**. Proportion of virus subtypes isolated from environmental samples during 2014–2018.

## Data Availability

The datasets used and analysed during the current study are available from the corresponding author on reasonable request.

## Abbreviations

AIVs: Avian influenza viruses
LPMs: Live poultry markets
PAM: Provinces, municipalities and autonomous regions
HA: Hemagglutinin
NA: Neuraminidase
SD: Standard deviation
