# Supplementary material for: Distribution of avian influenza viruses according to environmental surveillance during 2014–2018, China
Source: Infect Dis Poverty. 2021 May 6;10:60. doi: 10.1186/s40249-021-00850-3 (PMC8101199; doi:10.1186/s40249-021-00850-3)
Supplement: Supplementary file 1 — Additional file 1: Table S1. Primer and probe sequences used in the detection of influenza virus. Table S2. The average nucleic acid positivity rate of influenza A, H5, H7 and H9 in different provinces, municipalities and autonomous regions. Table S2-a, S2-b, S2-c, S2-d indicated the average nucleic acid positivity rate of influenza A, H5, H7 and H9 respectively. Table S3. The monthly nucleic acid positivity rate of influenza A, H5, H7 and H9 in environmental samples related poultry during 2014–2018. Table S4. Proportion of virus subtypes isolated from environmental samples during 2014–2018. [file 40249_2021_850_MOESM1_ESM.doc]

**Title:** Distribution of avian influenza virus in environmental surveillance during 2014-2018, China

**Authors:** Hong Bo , Ye Zhang , Libo Dong, Jie Dong ^,^ Xiyan Li , Xiang Zhao , Zi Li , Yuelong Shu , Dayan Wang

**Corresponding author:** Dayan Wang

**Affiliations:**

National Institute for Viral Disease Control and Prevention, Chinese Center for Disease Control and Prevention; WHO Collaborating Center for Reference and Research on Influenza; Key Laboratory for Biosafety, National Health Commission.

**Corresponding author email:** wangdayan@ivdc.chinacdc.cn.

**Supplementary materials**

**Table S1. Primer and probe sequences used in the detection of influenza virus.**

| **Targeted gene** | **Primer and probe sequences (from 5'-3')** |
| --- | --- |
| **M for FluA** | Forward primer: gaccratcctgtcacctctgac |
|  | Reverse primer: gggcattytggacaaakcgtctacg |
|  | Probe: FAM-tgcagtcctcgctcactgggcacg-BHQI |
| **HA for H5 subtype** | Forward primer: tggaaagtrtaaraaacggaacgt |
|  | Reverse primer: ygctagggarctcgccactg |
|  | Probe: HEX-tacccgcagtattcagaagaagc-BHQI |
| **HA for H7 subtype** | Forward primer: agaaatgaaatggctcctgtcaa |
|  | Reverse primer: ggttttttcttgtatttttatatgacttag |
|  | Probe: HEX -agataatgctgcattcccgcagatg-BHQI |
| **HA for H9 subtype** | Forward primer: caagctggaatctgarggaacttaca |
|  | Reverse primer: gcatctgcaagatccattggacat |
|  | Probe: FAM-cccagaacargaaggcagcaaaccccattg-BHQI |

**Table S2. The average nucleic acid positivity of influenza A, H5, H7 and H9 in different provinces, municipalities and autonomous regions. Table S2-a, S2-b, S2-c, S2-d indicated the average nucleic acid positivity rate of influenza A, H5, H7 and H9 respectively.**

**Table S2-a**

| No. | Provinces | Positivity rate of FluA(%) |
| --- | --- | --- |
| 1 | Beijing(BJ) | 0.00 |
| 2 | Tibet(XZ) | 0.00 |
| 3 | Liaoning(LN) | 1.11 |
| 4 | Heilongjiang(HLJ) | 1.34 |
| 5 | Tianjin(TJ) | 1.66 |
| 6 | Hebei(HE) | 1.77 |
| 7 | Shanghai(SH) | 2.36 |
| 8 | Jilin(JL) | 2.45 |
| 9 | Shanxi(SX) | 4.25 |
| 10 | Hainan(HaN) | 4.47 |
| 11 | Shaanxi(SN) | 7.35 |
| 12 | Inner Mongolia(NM) | 9.33 |
| 13 | Shandong(SD) | 9.51 |
| 14 | Ningxia(NX) | 11.06 |
| 15 | Sichuan(SC) | 13.20 |
| 16 | Henan(HA) | 13.49 |
| 17 | Jiangsu(JS) | 13.65 |
| 18 | Qinghai(QH) | 15.00 |
| 19 | Hubei(HB) | 16.95 |
| 20 | Anhui(AH) | 17.31 |
| 21 | Yunnan(YN) | 17.87 |
| 22 | Xinjiang(XJ) | 19.79 |
| 23 | Guangdong(GD) | 21.69 |
| 24 | Gansu(GS) | 25.96 |
| 25 | Chongqing(CQ) | 26.96 |
| 26 | Zhejiang(ZJ) | 30.85 |
| 27 | Guangxi(GX) | 37.66 |
| 28 | Jiangxi(JX) | 38.60 |
| 29 | Hunan(HN) | 43.55 |
| 30 | Guizhou(GZ) | 44.59 |
| 31 | Fujian(FJ) | 45.51 |
|  | average | 22.57 |

**Table S2-b**

| No. | Provinces | Positivity rate of H5(%) |
| --- | --- | --- |
| 1 | Beijing(BJ) | 0.00 |
| 2 | Shanxi(SX) | 0.00 |
| 3 | Shanghai(SH) | 0.00 |
| 4 | Tibet(XZ) | 0.00 |
| 5 | Heilongjiang(HLJ) | 0.07 |
| 6 | Jilin(JL) | 0.11 |
| 7 | Liaoning(LN) | 0.22 |
| 8 | Gansu(GS) | 0.23 |
| 9 | Tianjin(TJ) | 0.23 |
| 10 | Shaanxi(SN) | 0.26 |
| 11 | Hena(HA) | 0.28 |
| 12 | Hebei(HE) | 0.48 |
| 13 | Yunnan(YN) | 0.55 |
| 14 | Ningxia(NX) | 0.64 |
| 15 | Shandong(SD) | 1.29 |
| 16 | Hainan(HaN) | 1.58 |
| 17 | Inner Mongolia(NM) | 1.86 |
| 18 | Xinjiang(XJ) | 1.86 |
| 19 | Jiangsu(JS) | 2.12 |
| 20 | Sichuan(SC) | 2.17 |
| 21 | Hubei(HB) | 2.25 |
| 22 | Anhui(AH) | 3.24 |
| 23 | Guangdong(GD) | 3.24 |
| 24 | Zhejiang(ZJ) | 3.95 |
| 25 | Guizhou(GZ) | 5.20 |
| 26 | Fujian(FJ) | 7.50 |
| 27 | Guangxi(GX) | 7.75 |
| 28 | Qinghai(QH) | 8.12 |
| 29 | Hunan(HN) | 11.43 |
| 30 | Jiangxi(JX) | 13.78 |
| 31 | Chongqing(CQ) | 13.79 |
|  | average | 3.89 |

**Table S2-c**

| No. | Provinces | Positivity rate of H7(%) |
| --- | --- | --- |
| 1 | Beijing(BJ) | 0.00 |
| 2 | Guizhou(GZ) | 0.00 |
| 3 | Heilongjiang(HLJ) | 0.00 |
| 4 | Qinghai(QH) | 0.00 |
| 5 | Shanxi(SX) | 0.00 |
| 6 | Tianjin(TJ) | 0.00 |
| 7 | Tibet(XZ) | 0.00 |
| 8 | Yunnan(YN) | 0.00 |
| 9 | Chongqing(CQ) | 0.05 |
| 10 | Liaoning(LN) | 0.07 |
| 11 | Hainan(HaN) | 0.11 |
| 12 | Ningxia(NX) | 0.16 |
| 13 | Shanghai(SH) | 0.20 |
| 14 | Hena(HA) | 0.28 |
| 15 | Shaanxi(SN) | 0.44 |
| 16 | Hebei(HE) | 0.44 |
| 17 | Jilin(JL) | 0.62 |
| 18 | Gansu(GS) | 0.79 |
| 19 | Sichuan(SC) | 0.92 |
| 20 | Xinjiang(XJ) | 1.13 |
| 21 | Shandong(SD) | 1.66 |
| 22 | Inner Mongolia(NM) | 2.18 |
| 23 | Guangxi(GX) | 2.22 |
| 24 | Hubei(HB) | 2.25 |
| 25 | Anhui(AH) | 2.58 |
| 26 | Guangdong(GD) | 3.11 |
| 27 | Jiangxi(JX) | 3.54 |
| 28 | Hunan(HN) | 3.76 |
| 29 | Jiangsu(JS) | 4.92 |
| 30 | Fujian(FuJ | 8.05 |
| 31 | Zhejiang(ZJ) | 9.25 |
|  | average | 3.41 |

**Table S2-d**

| No. | Provinces | Positivity rate of H9 (%) |
| --- | --- | --- |
| 1 | Beijing(BJ) | 0.00 |
| 2 | Tibet(XZ) | 0.00 |
| 3 | Liaoning(LN) | 0.75 |
| 4 | Shanghai(SH) | 1.08 |
| 5 | Hebei(HE) | 1.16 |
| 6 | Heilongjiang(HLJ) | 1.28 |
| 7 | Tianjin(TJ) | 1.39 |
| 8 | Jilin(JL) | 2.11 |
| 9 | Hainan(HaN) | 2.71 |
| 10 | Shanxi(SX) | 4.16 |
| 11 | Inner Mongolia(NM) | 5.58 |
| 12 | Shandong(SD) | 5.75 |
| 13 | Sichuan(SC) | 6.47 |
| 14 | Shaanxi(SN) | 6.63 |
| 15 | Henan(HA) | 8.03 |
| 16 | Guizhou(GZ) | 8.29 |
| 17 | Jiangsu(JS) | 8.55 |
| 18 | Ningxia(NX) | 10.32 |
| 19 | Qinghai(QH) | 11.89 |
| 20 | Hubei(HB) | 12.92 |
| 21 | Guangdong(GD) | 13.06 |
| 22 | Zhejiang(ZJ) | 13.37 |
| 23 | Anhui(AH) | 13.50 |
| 24 | Yunnan(YN) | 15.27 |
| 25 | Xinjiang(XJ) | 17.48 |
| 26 | Chongqing(CQ) | 17.72 |
| 27 | Jiangxi(JX) | 21.01 |
| 28 | Hunan(HN) | 22.34 |
| 29 | Guangxi(GX) | 22.63 |
| 30 | Gansu(GS) | 24.92 |
| 31 | Fujian(FJ) | 27.07 |
|  | average | 13.07 |

**Table S3. The monthly nucleic acid positivity rate of influenza A, H5, H7 and H9 in environmental samples related poultry during 2014-2018.**

| **Year/Month** | **The monthly nucleic acid positivity rate (%)**  **of influenza A, H5, H7 and H9** | | | |
| --- | --- | --- | --- | --- |
|  | **FLUA** | **H5** | **H7** | **H9** |
| 2014/01 | 24.56 | 3.18 | 9.46 | 7.82 |
| 2014/02 | 22.65 | 7.34 | 4.76 | 8.08 |
| 2014/03 | 23.22 | 8.43 | 5.29 | 9.33 |
| 2014/04 | 19.65 | 6.48 | 3.37 | 8.63 |
| 2014/05 | 15.93 | 3.51 | 2.34 | 7.83 |
| 2014/06 | 15.23 | 2.19 | 2.10 | 9.73 |
| 2014/07 | 17.13 | 3.28 | 1.85 | 10.59 |
| 2014/08 | 14.17 | 2.63 | 0.30 | 9.85 |
| 2014/09 | 17.39 | 2.01 | 0.75 | 12.75 |
| 2014/10 | 21.57 | 5.47 | 2.38 | 14.96 |
| 2014/11 | 24.74 | 4.46 | 2.82 | 17.32 |
| 2014/12 | 32.36 | 6.15 | 3.13 | 20.89 |
| 2015/01 | 32.97 | 6.65 | 9.36 | 19.37 |
| 2015/02 | 28.08 | 5.61 | 7.64 | 13.64 |
| 2015/03 | 21.00 | 3.70 | 3.21 | 11.21 |
| 2015/04 | 23.48 | 5.24 | 2.11 | 15.28 |
| 2015/05 | 17.11 | 2.03 | 1.62 | 10.53 |
| 2015/06 | 17.08 | 2.48 | 1.62 | 10.35 |
| 2015/07 | 18.01 | 3.47 | 1.05 | 12.34 |
| 2015/08 | 18.50 | 3.75 | 0.88 | 10.40 |
| 2015/09 | 14.37 | 2.25 | 0.89 | 9.47 |
| 2015/10 | 19.00 | 3.11 | 1.95 | 12.36 |
| 2015/01 | 27.07 | 4.46 | 1.38 | 14.90 |
| 2015/12 | 28.55 | 6.12 | 2.89 | 16.87 |
| 2016/01 | 39.22 | 9.26 | 9.73 | 22.37 |
| 2016/02 | 33.56 | 6.78 | 6.05 | 15.43 |
| 2016/03 | 29.16 | 6.33 | 3.48 | 15.69 |
| 2016/04 | 24.41 | 3.77 | 3.92 | 11.56 |
| 2016/05 | 22.21 | 3.04 | 1.52 | 12.16 |
| 2016/06 | 17.90 | 1.62 | 2.20 | 9.86 |
| 2016/07 | 17.17 | 2.18 | 1.09 | 10.11 |
| 2016/08 | 18.87 | 2.68 | 1.51 | 10.94 |
| 2016/09 | 20.10 | 2.44 | 1.30 | 11.65 |
| 2016/10 | 21.03 | 2.17 | 2.40 | 13.01 |
| 2016/11 | 25.12 | 3.94 | 3.39 | 15.63 |
| 2016/12 | 31.57 | 3.41 | 9.39 | 19.14 |
| 2017/01 | 28.42 | 3.18 | 9.97 | 14.83 |
| 2017/02 | 13.80 | 1.30 | 5.20 | 7.38 |
| 2017/03 | 21.24 | 2.70 | 4.56 | 11.88 |
| 2017/04 | 19.35 | 2.07 | 4.22 | 9.75 |
| 2017/05 | 11.66 | 1.13 | 1.58 | 5.65 |
| 2017/06 | 14.52 | 1.74 | 2.37 | 8.77 |
| 2017/07 | 12.23 | 0.90 | 1.76 | 8.76 |
| 2017/08 | 12.93 | 2.14 | 0.50 | 8.75 |
| 2017/09 | 13.85 | 1.14 | 0.62 | 10.34 |
| 2017/10 | 18.09 | 2.66 | 0.97 | 11.82 |
| 2017/11 | 25.48 | 3.37 | 0.77 | 16.06 |
| 2017/12 | 25.65 | 4.28 | 0.95 | 17.67 |
| 2018/01 | 28.74 | 5.40 | 0.84 | 19.32 |
| 2018/02 | 23.32 | 4.38 | 0.78 | 14.99 |
| 2018/03 | 19.25 | 2.53 | 0.33 | 12.55 |
| 2018/04 | 20.84 | 2.81 | 0.27 | 13.93 |
| 2018/05 | 16.17 | 1.58 | 0.54 | 12.57 |
| 2018/06 | 15.16 | 2.69 | 0.00 | 11.31 |
| 2018/07 | 15.35 | 2.42 | 0.00 | 11.66 |
| 2018/08 | 15.17 | 2.69 | 0.08 | 11.09 |
| 2018/09 | 17.12 | 3.42 | 0.02 | 12.64 |
| 2018/10 | 21.19 | 4.87 | 0.07 | 15.50 |
| 2018/11 | 24.67 | 6.42 | 0.06 | 15.89 |
| 2018/12 | 27.49 | 5.56 | 0.14 | 17.18 |

**Table S4. Proportion of virus subtypes isolated from environmental samples during 2014-2018.**

| **Subtypes** | **Proportion of virus subtypes (%) during 2014-2018** | | | | | |
| --- | --- | --- | --- | --- | --- | --- |
|  |  |  |  |  |  |  |
|  | **2014** | **2015** | **2016** | **2017** | **2018** | **average** |
| H1N1 | 0.35 | 0 | 0 | 0 | 0 | 0.07 |
| H1N2 | 0.35 | 0 | 0 | 0.52 | 0 | 0.17 |
| H1N3 | 0 | 0 | 0 | 0 | 0.26 | 0.05 |
| H1N8 | 0.7 | 0 | 0 | 0 | 0 | 0.14 |
| H2N2 | 0 | 0 | 0 | 0 | 0.13 | 0.03 |
| H3N2 | 4.9 | 6.22 | 5.79 | 6.98 | 6.85 | 6.15 |
| H3N3 | 0.35 | 0.24 | 0.25 | 0.26 | 0.13 | 0.25 |
| H3N6 | 0 | 0.48 | 0 | 0 | 0 | 0.10 |
| H3N8 | 0.7 | 0.96 | 0 | 0 | 0.26 | 0.38 |
| H4N2 | 0.7 | 0.72 | 0.5 | 0.52 | 0 | 0.49 |
| H4N3 | 0.35 | 0.24 | 0 | 0 | 0 | 0.12 |
| H4N6 | 0 | 0 | 0 | 0.26 | 0.26 | 0.10 |
| H4N8 | 0.35 | 0 | 0 | 0.26 | 0 | 0.12 |
| H5N1 | 8.74 | 10.53 | 5.54 | 5.94 | 0.8 | 6.31 |
| H5N2 | 1.4 | 2.87 | 1.76 | 1.81 | 0.26 | 1.62 |
| H5N6 | 11.19 | 18.66 | 34.01 | 12.14 | 27.28 | 20.66 |
| H5N8 | 0.7 | 0 | 0 | 0.26 | 0 | 0.19 |
| H5N9 | 0 | 0.24 | 0.25 | 0.26 | 0 | 0.15 |
| H6N2 | 0.35 | 0 | 0 | 0 | 1.2 | 0.31 |
| H6N6 | 2.1 | 3.59 | 4.79 | 4.13 | 7.52 | 4.43 |
| H6N8 | 0 | 0 | 0.25 | 0 | 0 | 0.05 |
| H7N3 | 0 | 0 | 0 | 0.26 | 0 | 0.05 |
| H7N7 | 0 | 0.24 | 0 | 0 | 0 | 0.05 |
| H7N8 | 0 | 0 | 0 | 0.26 | 0 | 0.05 |
| H7N9 | 6.29 | 3.59 | 11.34 | 23 | 0.26 | 8.90 |
| H9N2 | 58.74 | 51.2 | 35.01 | 36.18 | 53.36 | 46.90 |
| H9N9 | 0.7 | 0 | 0 | 0 | 0 | 0.14 |
| H10N1 | 0 | 0 | 0.25 | 0 | 0 | 0.05 |
| H10N3 | 0 | 0 | 0.25 | 0 | 0 | 0.05 |
| H10N8 | 0.7 | 0.24 | 0 | 0 | 0..53 | 0.24 |
| H11N9 | 0.35 | 0 | 0 | 0 | 0 | 0.07 |
| Mixture | 0 | 0 | 0 | 6.97 | 0.94 | 1.58 |
